# Eosinophilic Granulomatosis With Polyangiitis Presenting With Oral Granuloma as the Initial Symptom: A Case Report

**DOI:** 10.3389/fmed.2022.842137

**Published:** 2022-05-10

**Authors:** Lirong Lin, Rongjie Yu, Luquan Zheng, Shuyu Gong, Jurong Yang

**Affiliations:** Department of Nephrology, The Third Affiliated Hospital of Chongqing Medical University (General Hospital), Chongqing, China

**Keywords:** eosinophilic granulomatosis with polyangiitis, case report, antineutrophil cytoplasmic antibody, ANCA-associated vasculitis (AAV), myeloperoxidase

## Abstract

Antineutrophil cytoplasmic antibody associated vasculitis includes granulomatosis with polyangiitis, eosinophilic granulomatosis with polyangiitis (EGPA), and microscopic polyangiitis. While EGPA has no specific symptoms, it usually presents as necrotizing vasculitis, eosinophil infiltration of the tissues and organs, and extravascular granuloma formation. Here, we report a patient who had a rare initial presentation of oral granuloma and had been previously misdiagnosed several times at other hospitals. He was finally diagnosed with EGPA and recovered after methylprednisolone and cyclophosphamide treatment. The disease EGPA can present with a rare initial presentation of oral granuloma, methylprednisolone, and cyclophosphamide can be a suitable choice of treatment.

## Introduction

Eosinophilic granulomatosis with polyangiitis (EGPA), a necrotizing systemic vasculitis involving small and medium-sized vessels, is also known as Churg–Strauss syndrome or allergic granulomatous vasculitis. The age of disease onset ranges from 40 to 60 years (1). More than 75% of EGPA patients present with sinusitis and asthma with associated eosinophilia (2, 3). Approximately 60% of patients have associated cardiac (4), renal, or neurological involvement (5). Patients with antineutrophil cytoplasmic antibody antineutrophil cytoplasmic antibody associated vasculitis (AAV) commonly have a poor prognosis. However, their 5-year survival rate can be improved to more than 70% after standardized immunotherapy (6). We report a patient who had a rare initial presentation of oral granuloma and had been previously misdiagnosed several times at other hospitals. He was finally diagnosed with EGPA and recovered after appropriate immunosuppressive treatments.

## Case Description

A 28-year-old male patient was admitted to the hospital after having oral pain for 1 month, skin ecchymosis with ulceration for 20 days, and abnormal renal function test results for 3 days. He had a medical history of chronic, untreated hepatitis-B for 18 years. One month prior to the admission, the patient began to experience oral pain. He was diagnosed with oral tuberculosis and received anti-tuberculosis treatment with rifampin and isoniazid, as well as appropriate analgesics when needed. He showed no improvement after 10 days of the treatment and developed ecchymosis and petechiae in both lower extremities. He was diagnosed with allergic purpura and received anti-allergic treatment with loratadine. Three days before coming to the hospital, he had shown no improvement with respect to oral pain or skin ecchymosis and had also developed foot ulcers. He returned to the hospital for further care.

**Figure 1:**
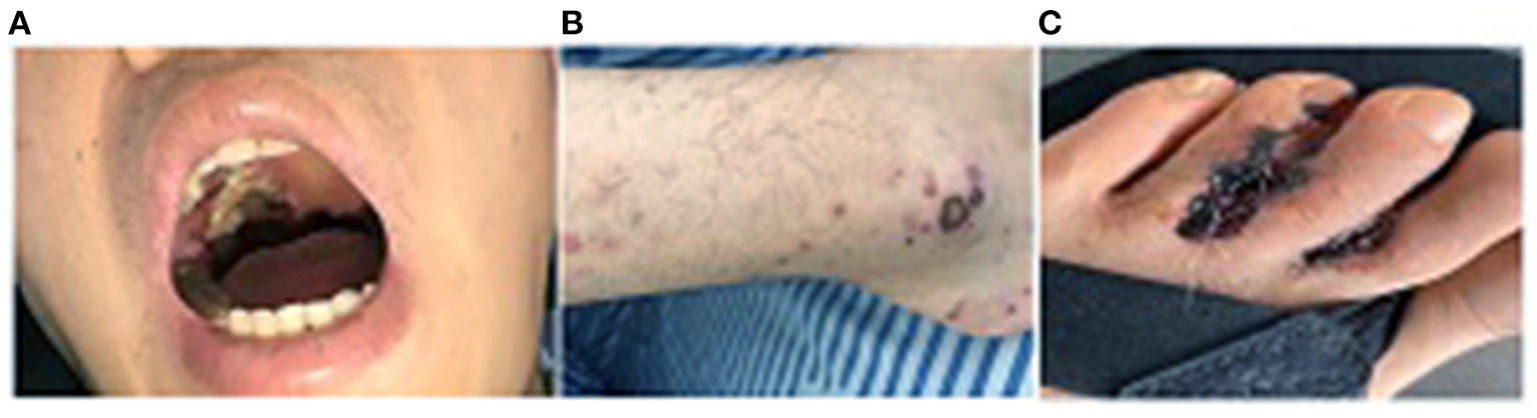
**(A)** A 5 cm × 5 cm grayish granuloma lesion in the oral palate; **(B)** Ecchymosis on bilateral lower extremities; **(C)** Skin ulcers with infection in the toes.

After hospital admission, a physical examination revealed an anemic appearance, with no edema in the face or lower extremities. The patient had coarse breath sounds with wet rales on lung auscultation. There was a 5 cm × 5 cm grayish–white necrotic tissue bulging from the right side of the oral palate (Figure 1A). The lower extremities showed ecchymosis with skin ulceration and necrosis in the left toes (Figures 1B,C). Laboratory test results were as follows: Red blood cell count, 2.56 × 10^12^/L; hemoglobin, 67 g/L; eosinophils, 21.2%; serum albumin, 31.9 g/L; alanine aminotransferase, 81.3 U/L; aspartate aminotransferase, 106 U/L; serum creatinine, 538 μmol/L; uric acid, 584 μmol/L; cholesterol, 6.37 mmol/L; low-density lipoprotein, 4.06 mmol/L; C-reactive protein, 34.4 mg/L; hepatitis-B surface antigen HBsAg positive, hepatitis-B virus DNA <10^2^, negative anti-nuclear antibody, and anti-double-stranded DNA antibody, normal serum immunoglobulin (Ig), IgA, IgM, and IgG, complement C3 and C4, and serum-free k- and λ-levels; normal serum anti-myeloperoxidase antibody level; and anti-proteinase 3 (PR3) antibody > 400 RU/ml. Urinary analysis showed urine protein 4+, urine microalbumin/creatinine 2,654 mg/g·Cr. A T-SPOT tuberculosis test and myocardial injury markers were negative. The oral ulcer smear was negative under acid-fast staining. Chest computerized tomography (CT) examination revealed bilateral multiple nodular shadows and pulmonary infection. Otolaryngoscopy suggested nasal mucosal erosion and necrosis. Sinus CT showed maxillary and ethmoid sinusitis. The Birmingham Vasculitis Activity Score was 27. Echocardiography showed mild mitral regurgitation. Electromyogram of extremities was unremarkable with normal sensory and motor functions.

## Diagnostic Assessment

**Figure 2:**
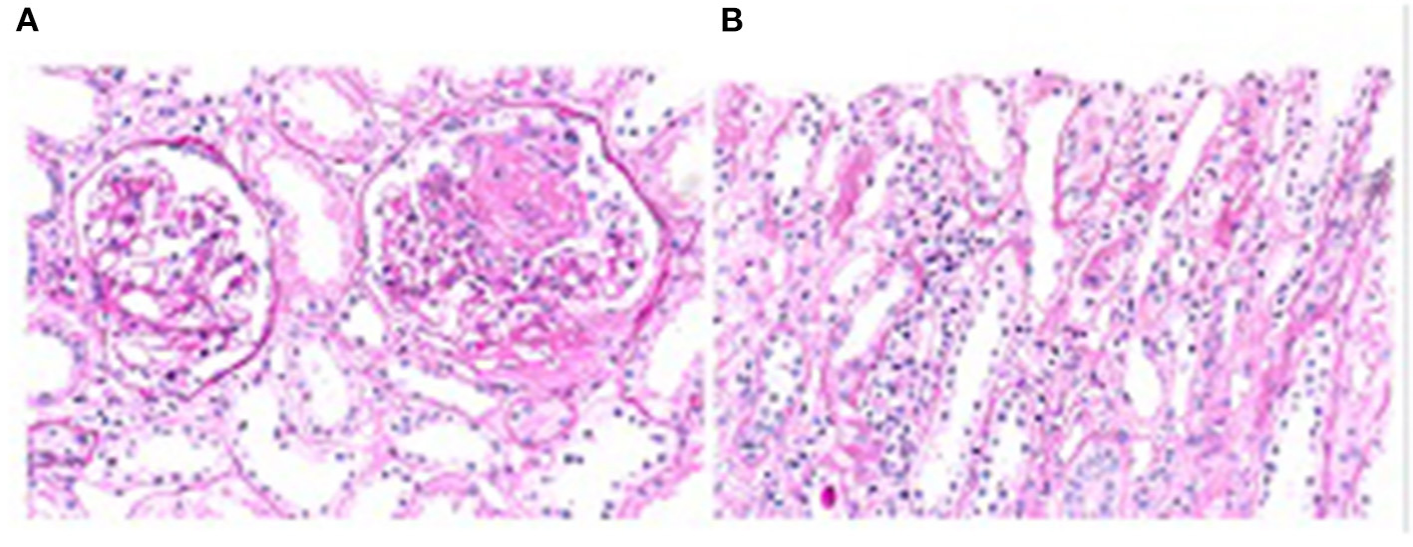
**(A)** Necrosis of segmental capillary loops, PAS, 200 ×; **(B)** Detachment of renal tubular epithelial cells and infiltration of interstitial inflammatory cells. PAS, 400 ×.

Pathological examination of the renal biopsy specimen showed that 2 of 14 glomeruli had global sclerosis. Two glomeruli had segmental necrosis of the capillary loops, with no cellular or fibrous crescents. There were extensive detachments of renal tubular epithelial cells and exposure of the basement membrane, with infiltration of small focal individual nucleated cells and eosinophils in the renal interstitium. The electron microscopic findings were consistent with the light microscopy findings. Immunofluorescence examination showed linear depositions of IgG(+) along the glomerular basement membrane, with negative staining for IgG1, IgG2, IgG3, IgG4, IgA, IgM, C3, C4, k, λ, and C1q. The final renal pathology diagnoses were acute tubulointerstitial kidney injury and antineutrophil cytoplasmic antibody (ANCA)-associated systemic vasculitis with kidney injury (nodular necrotic lesions) (Figures 2A,B).

Based on the 2016 treatment guidelines for vasculitis issued by the European League Against Rheumatism, we administered methylprednisolone 40 mg/day and cyclophosphamide 0.6 g intravenously for 10 days. The patient showed gradual improvement, including less oral pain, reduced size of skin ulcers, the disappearance of ecchymosis, and healing toe ulcers. His serum creatinine also decreased to 125 μmol/L.

**Figure 3:**
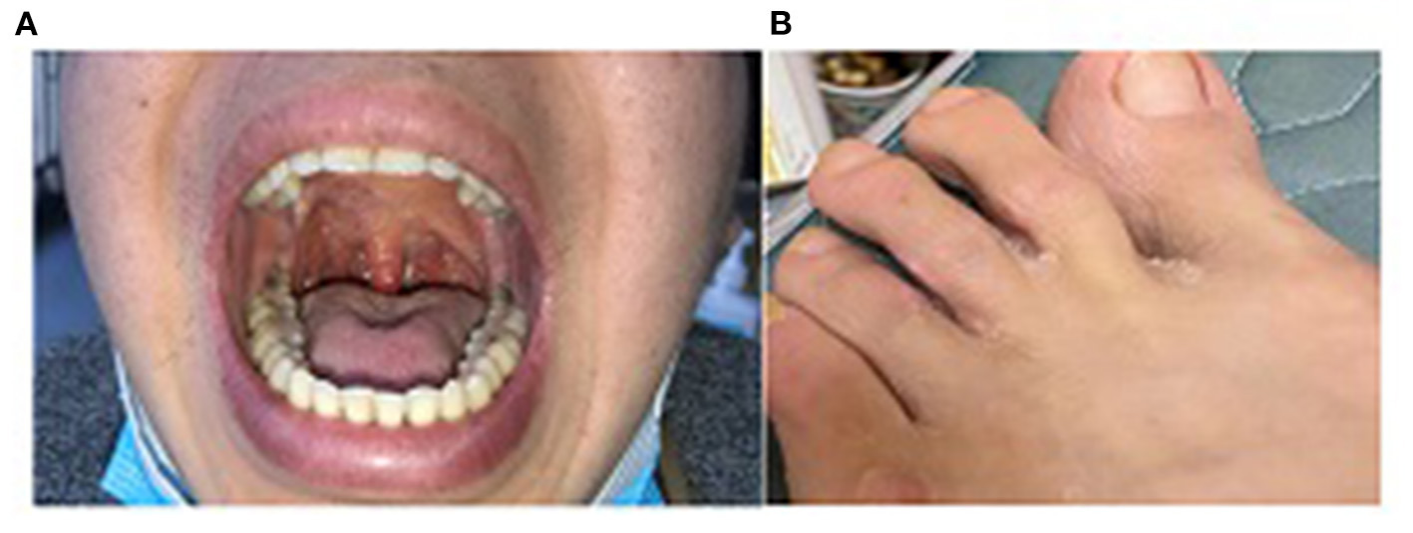
**(A)** Oral granuloma healed after treatment for 6 months; **(B)** Foot ulcer healed after treatment for 4 months.

We followed up with this patient every 1–2 months and performed CT scanning and laryngoscopy at the second follow-up. Results suggested that pulmonary nodules dissipated and ethmoid sinusitis improved. There had been a total of eight follow-up visits to date. His symptoms have significantly improved. Furthermore, the vasculitis score decreased to 4. Serum PR3 was normalized and anemia was corrected. Serum creatinine and estimated glomerular filtration rate returned to normal. Urine microalbumin/creatinine decreased to <1,000 mg/g·Cr. Oral granuloma, foot ulcers, and nose and throat lesions were also healed (Figures 3A,B).

## Discussion

Our patient presented with previously unreported oral granuloma as the first complaint. This atypical early presentation may have led to misdiagnosis as oral tuberculosis. With disease progression, this patient gradually developed more lesions in the skin, mucosa, lungs, kidneys, ear, nose, and throat, which led us to consider an immune disease with multisystem and multiorgan injuries.

According to 2021 Kidney Disease: Improving Global Outcomes (KDIGO) guidelines suggested, plasma exchange should be considered for patients with SCr > 5.7 mg/dl (500 mmol/L) requiring dialysis, for patients with rapidly increasing SCr, and for patients with diffuse alveolar hemorrhage who have hypoxemia (7). The patient also had abnormal renal function as shown in the laboratory test, which could easily lead to misdiagnosis and mismanagement. Patients with ANCA-associated systemic vasculitis tend to have renal injuries, which present as abnormal renal function and crescentic and capillary loop necrosis. Initially, we considered that our patient had acute or chronic renal failure due to ANCA-mediated crescentic glomerulonephritis. However, the renal pathological examination did not support this initial diagnosis. The renal pathological changes not only included ANCA-mediated nodular necrotic lesions but also acute tubulointerstitial kidney injury, which might have been caused by previous analgesic intake and anti-TB treatment. Considering the renal pathology report, we lowered his vasculitis activity score from 27 to 24.

After confirming the diagnosis of this patient, we reviewed the 2021 KIDGO guidelines on the treatment of renal injury in ANCA-associated systemic vasculitis (7), the 2016 treatment guidelines for the vasculitis from the European League Against Rheumatism (8), and the stratified treatment strategy from the EGPA Five-Factor Score (9). We decided to treat him with an induction therapy regimen including glucocorticoids at 1.0 mg/kg/day and cyclophosphamide at 0.6 g/month. Glucocorticoids were administered at a dose of 1.0 mg/kg/day for 1 month, after which they were gradually tapered off. During the maintenance therapy, glucocorticoids at 5–15 mg/day and cyclophosphamide at 0.6 g were given for 2 months. The patient's symptoms gradually improved during the 12-month follow-up period.

In summary, our patient had a confirmed diagnosis and his symptoms significantly improved after glucocorticoid and cyclophosphamide treatments. However, we were unable to make an accurate pathological diagnosis of oral granuloma because the patient declined oral biopsy. As such, we performed a laboratory and imaging examinations to exclude oral granulomas caused by tuberculosis, fungal infection, or thrombotic disease. However, the clinical improvement he showed after treatment suggested a close association between oral granuloma and primary disease. We learned two important lessons from the patient's presentations: (1) Performing renal biopsy and pathological examination is important in patients with ANCA-related systemic vasculitis and renal involvement because these patients can sustain renal injury from other causes mediated by factors unlike those of ANCA; (2) performing biopsy and pathological examination early is also important in patients with skin and mucosal granulomatous lesions to establish a clear diagnosis and prevent delays in treatment.

## Data Availability Statement

The original contributions presented in the study are included in the article/supplementary material, further inquiries can be directed to the corresponding author.

## Author Contributions

LL and SG collected all the data, carried out the analysis of the patient's clinical course and outcome, and were also involved in drafting the manuscript. LZ and RY were responsible for the pathology images and legends. JY established the diagnosis and revised and approved the final manuscript. Each author contributed important intellectual content during the drafting and revision of the manuscript. All authors have read and approved the final version of the manuscript to be published.

## Funding

This work was supported by grants (cstc2019jscx-msxmX0166) from the Basic, Frontier Research Program of Chongqing and the Chongqing Talent Program Project (cstc2021ycjh-bgzxm0090) and Scientific Research Incubation Project of the Third Affiliated Hospital of Chongqing Medical University (KY20078).

## Conflict of Interest

The authors declare that the research was conducted in the absence of any commercial or financial relationships that could be construed as a potential conflict of interest.

## Publisher's Note

All claims expressed in this article are solely those of the authors and do not necessarily represent those of their affiliated organizations, or those of the publisher, the editors and the reviewers. Any product that may be evaluated in this article, or claim that may be made by its manufacturer, is not guaranteed or endorsed by the publisher.

## Abbreviations

ANCA: antineutrophil cytoplasmic antibody
AAV: antibody associated vasculitis
GPA: granulomatosis with polyangiitis
EGPA: eosinophilic granulomatosis with polyangiitis
MPA: microscopic polyangiitis
PR3: anti-proteinase 3.
